# Functionalized GD2 Electrochemical Immunosensor to Diagnose Minimum Residual Disease of Bone Marrow in Neuroblastoma Effectively

**DOI:** 10.3390/bios13100920

**Published:** 2023-10-10

**Authors:** Chong Chen, Chang Hu, Baixun He, Yongchang Bai, Feng He, Shuang Li, Cherie S. Tan

**Affiliations:** 1 Academy of Medical Engineering and Translational Medicine, Tianjin University, Tianjin 300072, China; chongchen@tmu.edu.cn (C.C.); chang_hu@tju.edu.cn (C.H.); baixun_he@tju.edu.cn (B.H.); baiyongchang@tju.edu.cn (Y.B.); heaven@tju.edu.cn (F.H.); 2 Department of Clinical Laboratory, Tianjin Medical University Cancer Institute and Hospital, Tianjin 300060, China

**Keywords:** neuroblastoma, bone marrow, GD2, electrochemical sensor, graphene/AuNPs

## Abstract

Neuroblastoma (NB) is known as the “king of childhood tumors” due to its highly metastatic, recurrence-prone, and difficult-to-treat characteristics. International Neuroblastoma Risk Grading Group (INRG) has recommended GD2, a disialoganglioside expressed on neuroectodermal tumor cells, as the target for detecting minimal residual disease in bone marrow metastases of high-risk neuroblastoma in children. Therefore, accurately identifying GD2-positive cells is crucial for diagnosing children with high-risk NB. Here, we designed a graphene/AuNP/GD2 Ab-functionalized electrochemical biosensor for GD2 detection. A three-electrode system was processed using a screen-printed technique with a working electrode of indium tin oxide, a counter electrode of carbon, and a reference electrode of silver/silver chloride. Graphene/AuNPs were modified on the indium tin oxide electrode using chronoamperometric scans, and then, the GD2 antibody was modified on the biosensor by electrostatic adsorption to achieve sensitive and specific detection of GD2-positive cells in bone marrow fluid. The results showed that a graphene/AuNP/GD2 Ab-functionalized electrochemical biosensor achieved GD2-positive cell detection in the range of 10^2^ cells/mL~10^5^ cells/mL by differential pulse voltammetry. Bone marrow fluid samples from 12 children with high-risk NB were retained for testing on our biosensor and showed 100% compliance with the clinical application of the gold-standard immunocytochemical staining technique for detecting GD2-positive cells qualitatively. The GD2-based electrochemical assay can accurately detect children with high-risk NB, providing a rapidly quantitative basis for clinical diagnosis and treatment.

## 1. Introduction

Neuroblastoma (NB) originates from the sympathetic ganglion or bilateral adrenal glands and is the most common extracranial solid tumor in childhood, having high metastasis, recurring easily, and being of the refractory type. Such clinical characteristics are inevitably determined by their unique molecular biology [1,2,3,4]. Neuroblastoma has significant clinical and biological heterogeneity, with some children having spontaneous regression without treatment and others having poor prognosis and extensive metastasis despite effective multimodal therapy [5]. The long-term survival rate of children with NB in the high-risk group is less than 50% despite aggressive multimodal therapy (radiotherapy, surgery, autologous stem cell transplantation, GD2 (disialoganglioside, GD2) monoclonal antibody, or maintenance therapy with retinoic acid) [4,6,7,8,9,10,11]. Studies have shown that children with NB in the high-risk group are most likely to develop bone marrow metastasis, and children with NB in the high-risk group are highly susceptible to recurrence because of minimal residual disease (MRD) in the bone marrow [12,13,14,15,16,17,18,19]. Bone marrow-associated neuroblastoma detection is crucial in diagnosing metastasis and recurrence in children with high-risk NB [2,3,8,13,20,21,22,23,24,25]. However, there is a lack of consensus on the standard determination of NB bone marrow recurrence and metastasis globally [14,15,16,26], and the existing diagnostic techniques cannot solve the critical clinical challenges in children with high-risk NB.

GD2 is widely expressed on the surface of neural tumor cells, in the microenvironment of tumor cells, and in the peripheral blood circulation of patients [27,28,29,30]. These GD2 molecules are involved in various cellular biological processes, such as acting as cell surface receptors, participating in intercellular signaling, and regulating cell cycle and cellular activity. It has been shown that neuroblastoma cells express higher levels of GD2 molecules on the cell surface than normal neural tissues [30,31]. GD2 may be a biomarker for differential diagnosis of NB cell surfaces versus other tumors, and it is also by far the most influential star target for high-risk NB [31]. It has been found that peripheral circulating GD2 expression is closely associated with disease progression in high-risk NB, as well as with its malignancy, the main reason for which may be related to the involvement of GD2 in neuroblastoma metastasis [30]. Recently, it has been applied to the clinical treatment of NB, and accurate detection of GD2 expression in NB is of great clinical value for clinical aid in diagnosing and monitoring recurrence [31].

Compared with normal neural tissues, neuroblastoma cells express high levels of disialoganglioside GD2. Even though melanoma, primitive neuroectodermal tumors, and osteosarcoma cells also express high levels of GD2 molecules on their surfaces, GD2 is still applied as the clinical therapeutic target for high-risk neuroblastoma [11,27,28,29,30,31]. NB bone marrow metastases are closely associated with disialoganglioside GD2, and the malignancy of NB bone marrow metastases (number of neuroblastomas, cell malignancy, and regression) can be monitored by GD2 activity. In 2009, the International Neuroblastoma Risk Grading Group (INRG) established a standard clinical method to detect microscopic lesions in children with NB, including immunocytochemical staining for GD2 antigen on the cell surface and real-time fluorescence quantitative PCR for tyrosine hydroxylase, a critical enzyme in GD2 synthesis [11,18,19,32]. There is a consensus on using GD2 as a precise diagnostic technique for monitoring bone marrow metastasis and MRD recurrence in children with high-risk NB. However, the existing diagnostic technical tools are based only on morphological classification techniques of bone marrow smears, and the sensitivity of morphological classification techniques is limited and unreliable. Moreover, the number of infiltrating tumor cells cannot be accurately quantified, and tumor cell counts below 0.1% are barely detectable by conventional cytomorphology [1,12,30,33,34]. Several studies have confirmed the sensitivity of immunocytochemical staining methods based on GD2 targeting in the detection of microscopic lesions in children with NB up to 0.001%, which could effectively complement the existing bone marrow smear morphological assays and provide laboratory evidence for the targeted use of GD2 drugs in children with high-risk NB [12,27,29,34,35,36].

Along with the rapid development of nanomaterials technology, there has been an improvement in tumor diagnostic-related techniques [37]. Among various organic and inorganic nanoparticles, gold nanoparticles (AuNPs) have unique physical, chemical, and biological properties that have been applied in tumor diagnostics [38]. Researchers typically combine adapters and AuNPs to detect tumor-specific biomarkers, and applying AuNPs to amplify electrochemical or optical signals can make the immunosensor more sensitive [39,40,41]. AuNPs have uniquely demonstrated physical and chemical properties to bind signaling compounds (fluorescent dyes [39], antibodies [42], aptamers [43], redox markers [44], or other chemical modifications with straightforward methods [45,46,47]). AuNPs also have a large bulk surface area for coupling biologically recognized fragments (e.g., nucleic acid aptamers, proteins, antibody fragments, or peptides), further enhancing target-specific therapeutic diversity and offering the possibility of new techniques for more tumor diagnostics [41,44,48]. Graphene is a two-dimensional nanomaterial with a high specific surface area, high electrical conductivity, a fast electron transfer process, good mechanical strength, and strong adhesion to metal/metal oxide nanoparticles [42,49]. Graphene-based nanocomposite materials find widespread applications in electrochemical sensing detection, owing to the ability to promote charge transfer on electrode surfaces [41,50,51,52].

The clinically applied GD2 immunocytochemical staining technique is cumbersome to operate, requires a long time to detect, requires a high level of operator proficiency, is prone to false-positives, and cannot meet diagnostic needs. Therefore, we developed a particular graphene/AuNP/GD2 Ab-functionalized electrochemical biosensor to sensitively and accurately detect GD2-positive cells in bone marrow fluid and achieve a rapid diagnosis of minimum residual disease.

## 2. Materials and Methods

### 2.1. Chemicals and Materials

Indium tin oxide (ITO) is a film on a glass plate that was used as a working electrode, synthesized by the mechanical force chemistry method [53]. Chemicals for the electrochemical reduction of AuNPs included sodium tetrachlorate (III) dihydrate (NaAuCl_4_·2H_2_O) and sodium sulfate (Na_2_SO_4_). Graphene dispersion was obtained from XFNANO (Nanjing, China). Chloroauric acid was obtained from Sigma-Aldrich (WI, USA). GD2 Ab (clone: 14.G2a) was obtained from Santa Cruz Biological Corporation (TX, USA). Phosphate-buffered saline (PBS, 0.01 M, pH = 7.4) and fetal bovine serum were obtained from Standard Information Network (Shanghai, China). The reference electrode was silver/silver chloride (Ag/AgCl), and the counter electrode was carbon.

### 2.2. Electrochemical Three-Electrode Fabrication

Firstly, the base of the electrode sheet with a length of 20 mm and width of 8.5 mm was made on ITO substrate; a circle with a diameter of 4 mm was left as the working electrode at the middle position of 12 mm from the bottom, and the rest was covered with a water barrier layer to prevent the working electrode solution from flowing out. Secondly, the working and reference electrodes were screen-printed using a screen-printing process with a 1 mm Ag/AgCl coating thickness and carbon-coated electrode surfaces. To co-center with the working electrode, an inner diameter of 5.8 mm and an outer diameter of 7.4 mm were applied to stabilize the three-electrode working system. The working electrode was indium tin oxide (ITO), synthesized by the mechanical force chemistry method. Chemicals for the electrochemical reduction of AuNPs included sodium tetrachlorate (III) dihydrate (NaAuCl_4_·2H_2_O) and sodium sulfate (Na_2_SO_4_). GD2 Ab was obtained from Santa Cruz Biolo2.2 Electrochemical three-electrode fabrication.

### 2.3. Graphene/AuNP Modification

A total of 1 mg/mL of graphene dispersion was mixed with 1% chloroauric acid in equal volume; 100 μL was added dropwise onto the fabricated ITO electrode; and the nanographene was modified on the electrode using the chronoamperometry method, in which the negative potential E = −0.6 V was set, with a deposition time at 40 s.

### 2.4. Graphene/AuNP/GD2 Ab-Functionalized Electrode Fabrication

**Figure 1:**
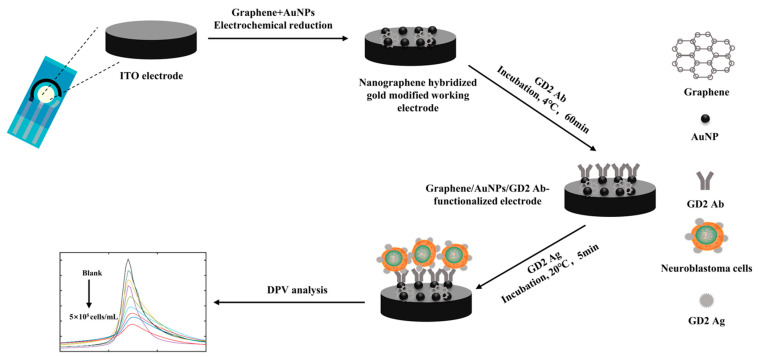
Schematic diagrams of the electrochemical sensing platform.

GD2 Ab was deposited into the prepared nanographene hybridized gold-modified electrode through electrostatic adsorption. Then, 100 μL of GD2 antibody at a concentration of 1 mg/mL was added dropwise onto the previously prepared modified electrode, and the excess non-adsorbed antibody was rinsed off with pure water after resting for one hour at 4 °C. Finally, the GD2 antibody/nanographene hybridized gold-modified ITO electrode was made for the assay (Figure 1).

Graphene/AuNP/GD2 Ab-functionalized electrodes work as follows. The working electrode was indium tin oxide (ITO) synthesized by mechanically forceful chemical methods. Chemicals used to reduce AuNPs electrochemically include sodium tetrachloride (III) dihydrate (NaAuCl_4_·2H_2_O) and sodium sulfate (Na_2_SO_4_). Then, GD2 antibody was applied to bind the working electrode, which generated a characteristic electrochemical peak under the DPV measurement. When cells were attached to the working electrode via antigen–antibody interaction, it altered the resistance and capacitance of the working electrode, resulting in decreased DPV response.

### 2.5. Cell Culture

The GD2-positive cells used in this study were IMR32 human NB cells. The cells were cultured at 37 ℃ using 90% Dulbecco’s modification of Eagle’s medium (DMEM), basal medium (Merk Life Science S.r.l., Milan, Italy), and 10% fetal bovine serum (Merk Life Science S.r.l., Milan, Italy) with 1% penicillin/streptomycin/amphotericin (Merk Life Science S.r.l., Milan, Italy) in a humidified environment containing 5% CO_2_. The medium was changed twice weekly, and cells were passaged when 80% confluence was reached.

### 2.6. Detection of the Proportion of GD2-Positive Cells in Bone Marrow Samples of Patients with High-Risk NB

Firstly, single-nucleated cells from bone marrow fluid were collected using density gradient centrifugation and then resuspended using 100 µL of DMEM to obtain bone marrow aspirate single-nucleated cell resuspension solution. A total of 100 µL of DMEM buffer or 100 µL of bone marrow single-nucleated cell resuspension solution was added to the functionalized electrode separately to obtain the differential pulse voltammetry (DPV) on the functionalized electrode, with a set initial voltage of −0.4 V, termination voltage of 0.4 V, increment of 0.005 V, amplitude of 0.050 V, pulse width of 0.05 s, and period of 0.5 s. Three scans of each electrode per concentration were performed. The difference (∆I) between the peak current of the two DPV measurements was plotted against the logarithm of the concentration of GD2-positive cells (IMR32 human NB cells), resulting in a linear correlation equation. The concentrations of GD2-positive cells in the patient samples were calculated based on the fitted curve.

### 2.7. Immunocytochemical Methods for Diagnosis of MRD of Bone Marrow in High-Risk NB

Firstly, freshly prepared sodium citrate anticoagulated bone marrow fluid was diluted with saline in equal proportion, then carefully added to the liquid surface of a lymphocyte separation solution, and then centrifuged with a density gradient (1000 rpm, 5 min) to separate the white flocculent layer, i.e., the single-nucleated cell layer, and secondly centrifuged (1000 rpm, 5 min) to obtain the single-nucleated cells and counted to prepare slides for examination, fix the cells with formalin liquid, incubate with GD2 antibody for 40 min, incubate with SAP for 15 min, develop color with AP-Red at room temperature for 30 min, re-stain with hematoxylin, seal the slides, and finally count the total number of GD2-positive cells under the microscope. The reporting pattern was the number of GD2-positive cells per million cells while determining the presence of MRD in the bone marrow of children with high-risk NB.

### 2.8. Flow Cytometry for Diagnosis of MRD of Bone Marrow in High-Risk NB

Firstly, freshly prepared sodium citrate anticoagulated bone marrow fluid was diluted equivalently with saline and carefully added to the liquid surface of the lymphocyte separation solution. The white flocculent layer, the single-nucleated cell layer, was separated by density gradient centrifugation (1000 rpm, 5 min), and a cell counting plate counted the cells in this layer. Again, 100 μL of single-nucleated cells (about 1 × 10^5^ cells) was added with 5 μL each of flow antibody (CD45, CD56, CD81, and GD2 antibodies), incubated for 30 min with protection from light, eluted by adding 1 mL of PBS, and finally resuspended with 300 μL of cell staining buffer for flow cytometry detection; CD45^−^, CD56^+^, CD81^+^, and GD2^+^ cells were NB cells, which were counted by flow cytometry to determine the presence of MRD in the bone marrow.

## 3. Results and Discussions

### 3.1. Graphene/AuNP/GD2 Ab-Functionalized Electrode Characterization

**Figure 2:**
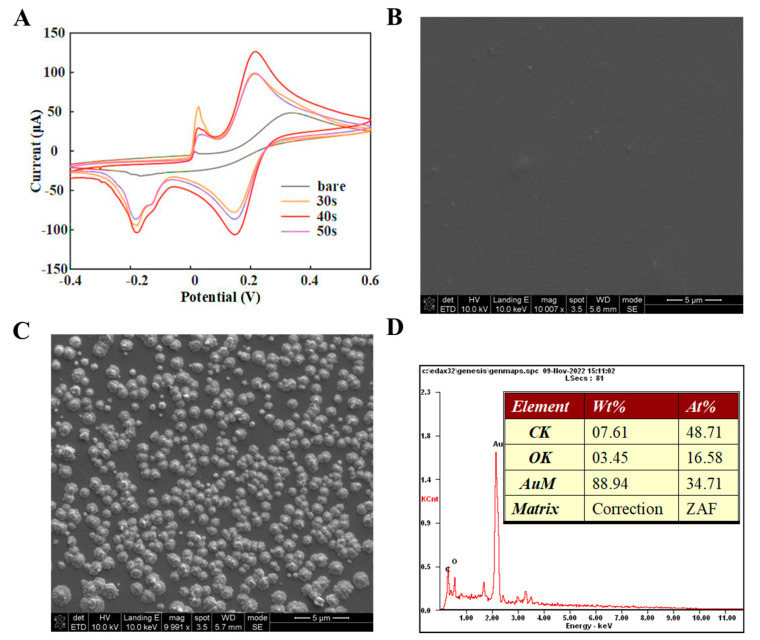
Surface modifications on the graphene/gold nanoparticles (AuNP) functionalized electrode. (**A**) Electrochemical deposition of nanocomposites on the electrode surface; (**B**,**C**) Scanning electron microscope (SEM) spectra of the bare indium tin oxide (ITO) and nanocomposite−modified electrodes, respectively; (**D**) Energy dispersive spectroscopy (EDS) spectrum of the nanocomposite−modified electrodes.

The electrochemical properties of the nanographene hybrid gold-modified electrode were characterized using cyclic voltammetry, at 50 mV/s. The bare electrode showed an oxidation peak at 0.338 V with a value of 48.5 μA. The peak current was 97.9 μA at voltage 0.216 V when deposited at t = 30 s. The peak current was 126.3 μA at voltage 0.216 V when deposited at t = 40 s. When deposited at t = 50 s, the peak current at voltage 0.214 V was 99.0 μA. The deposition time was chosen to be 40 s (Figure 2A).

The dispersion and distribution of the nanocomposite on the electrode were characterized by SEM, as shown in Figure 2C. As the functionalization of the self-assembly proceeded, more active edges and surfaces were created. In addition to morphologically observing the self-assembly process of the electrode, an EDS analysis was used to illustrate the distribution of elements on the electrode. Figure 2D shows the EDS spectra of the graphene/AuNP/GD2 Ab-functionalized electrode, where both C and Au elements are uniformly distributed on the electrode surface. These results elucidated the relevant properties of electrodes modified with nanocomposites regarding electrochemical analysis and micromorphological distribution and provided technical support for subsequent biosensing.

### 3.2. GD2-Positive Cell Biosensing

**Figure 3:**
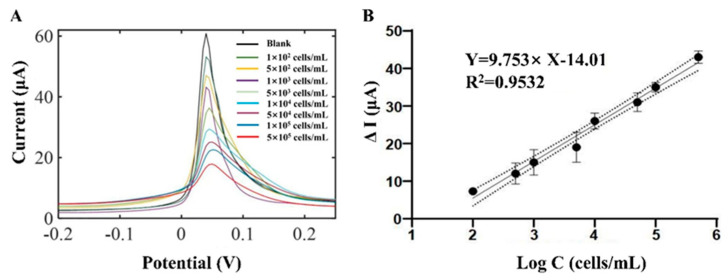
GD2-positive cells biosensing. (**A**) Differential pulse voltammetry (DPV) curves of GD2^−^-positive cell detection from 10^2^ to 5 × 10^5^ cells/mL in dulbecco’s modified eagle’s medium (DMEM). (**B**) Linearly dependent curve between the concentration and the current difference (ΔI = I_DMEM_ − I_GD2+_).

Differential pulse voltammetry (DPV) was applied to evaluate the performance of the biosensor on GD2-expressing positive cells, IMR32. The DPV measurements are initial voltage −0.4 V, termination voltage 0.4 V, increment 0.005 V, amplitude 0.05 V, pulse width 0.05 s, period 0.5 s, and sampling frequency 4 Hz. The graphene/AuNP-GD2 Ab-functionalized electrode generated a characteristic electrochemical peak under the DPV measurement at 0.05 V. When GD2-expressing positive cells were attached to the working electrode via antigen–antibody interaction, they altered the resistance and capacitance of the working electrode, resulting in decreased DPV response. As shown in Figure 3A, the DPV peak currents decreased gradually as GD2-positive cell concentrations increased from 10^2^ cells/mL to 10^5^ cells/mL. The peak current differences ΔI, ΔI = I_DMEM_ − I_GD2+_, were plotted against the logarithm of the concentration of GD2-positive cells. The fitting equation is ∆I = 9.753 × lg[GD2] − 14.01 with an R-squared correlation factor of 0.9532, and the limit of detection (LOD) was 10^2^ cells/mL. Compared with immunocytochemical staining techniques that require secondary antibodies, these gold-modified electrodes achieved sensitive detection of GD2-positive cells, reducing the underlying impedance with high detection accuracy and a more comprehensive detection range.

### 3.3. Bone Marrow Sample Detection in Children with NB

**Figure 4:**
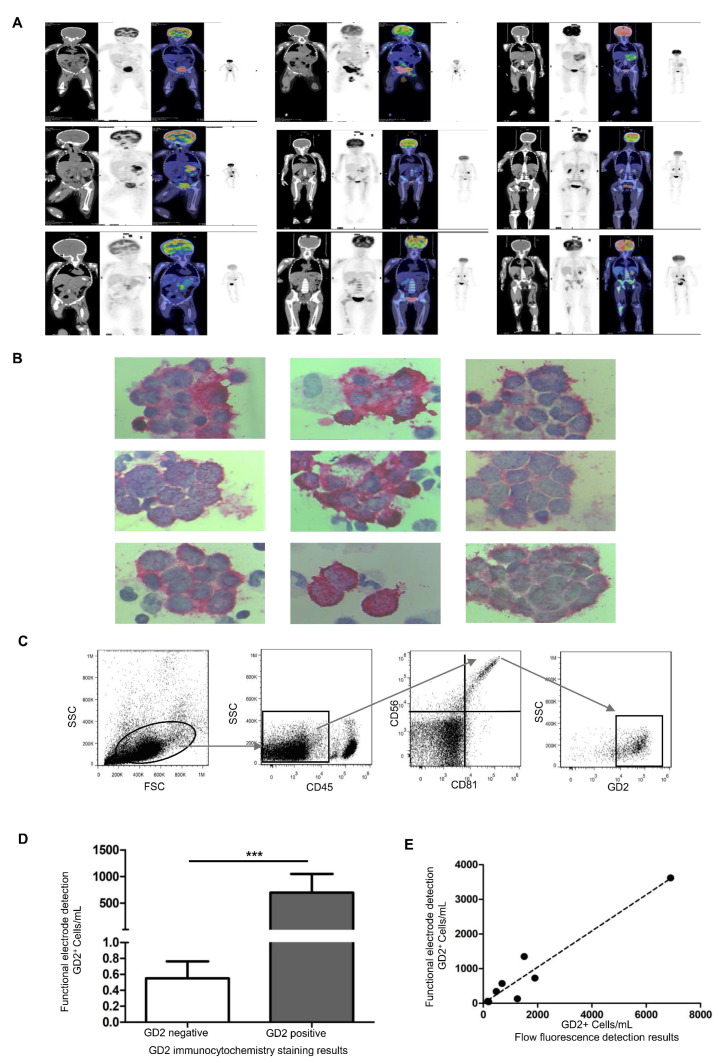
Bone marrow sample detection in children with neuroblastoma (NB). (**A**) Positron emission tomography-computedtomography (PET-CT) images suggest bone marrow metastasis; (**B**) immunocytochemical staining results of bone-marrow minimal residual disease (MRD); (**C**) flow-fluorescence detection suggests GD2-positive bone marrow MRD; (**D**) comparison of GD2-positive cell concentrations in children with different GD2-expressing NB cells detected by graphene/AuNP/GD2 Ab-functionalized electrode (*** *p* < 0.01); (**E**) correlation of functional electrode detection of GD2-positive cell concentration with the results of a flow-fluorescence technique, with the slope of 0.5227.

A total of nine children, six boys and three girls, were included in this study. All enrolled children had been pathologically diagnosed with NB with poor differentiation and high risk. The bone marrow samples used in the study were obtained at Tianjin Medical University Cancer Institute & Hospital, a residual sample used clinically to diagnose small residual lesions in the bone marrow. All enrolled patients underwent PET-CT screening, and the PET-CT results of the positive patients showed an uneven increase in systemic bone marrow metabolism but did not exclude bone marrow metastasis (Figure 4A). In addition, bone marrow nucleated cells were stained using the GD2 immunocytochemical staining technique, and NB metastatic cancer cells were explicitly stained red (Figure 4B), showing that all patients had GD2-positive cells in their specimens. Furthermore, bone marrow specimens from GD2-positive patients were also counted using flow cytometry (Figure 4C). The presence of GD2-positive cells in the bone marrow was also analyzed using functional electrodes, and the concentration of GD2-positive cells was calculated using a linear regression analysis model. Here, ΔI = I_DMEM_ − I_sample_, where I_DMEM_ is the peak current of DPV in artificial DMEM only and I_sample_ is the peak current of DPV in the bone marrow of children with NB. The peak current of DPV in bone marrow fluid from the enrolled nine GD2-positive patients and ΔI was introduced into the linear regression analysis model (ΔI = 9.753 × LogC − 14.01) to calculate GD2-positive cell concentration. The results showed that the ability of functional electrodes to determine the presence of GD2-positive cells in bone marrow was consistent with the GD2 immunocytochemical staining technique (Figure 4D); the linear regression analysis model to calculate the GD2-positive cell concentration was positively correlated with the number of GD2-positive cells by flow assay (*p* < 0.001), and the correlation coefficient was 0.932 (Figure 4E).

## 4. Discussion

Traditional screening techniques for neuroblastoma include peripheral blood tumor markers, ultrasound of the abdomen, PET-CT, and ECT, which are noninvasive and can be used multiple times. However, they cannot diagnose small residual lesions and monitor bone marrow recurrence in children with high-risk neuroblastoma. PET-CT is an essential method for the diagnosis of neuroblastoma in children, whether bone marrow MRD or not, and it is also a diagnostic technique recognized by pediatric specialists as a noninvasive method for clinical application of screening for bone marrow metastasis. However, it is expensive and has the disadvantages of false-positive and false-negative results. Bone marrow aspiration and biopsy are the gold standard for diagnosing bone marrow metastasis and neuroblastoma recurrence and are also the recommended clinical diagnostic basis [54,55,56]. In this paper, an immunosensor was designed for GD2, which is an important detection target of neuroblastoma, and the corresponding GD2 antibody was modified on the sensor surface according to the specific binding of antigen and antibody in order to improve the detection of GD2 molecules on the surface of neuroblastoma.

Research has shown that immunosensors can be applied to detecting MRD in lung cancer [57]. This study found that the sensitivity of the clinically applied immunocytochemical staining technique for the diagnosis of bone marrow MRD was 0.001%. In contrast, this immunosensor’s detection limit was 0.0002%, which improves the ability to detect tiny residual lesions and more accurately determine the presence or absence of bone marrow metastasis or recurrence. Meanwhile, the whole diagnostic process of immunocytochemical staining takes at least four hours, while our immunosensor can be completed in only two hours. Precision diagnosis of GD2 is crucial for neuroblastoma patients’ pre-treatment evaluation, formulation of treatment plans, recurrence detection, and prognosis assessment. This immunosensor significantly shortens the waiting time for the clinic and provides laboratory evidence for the clinic to make the correct decision promptly. This immunosensor significantly reduces clinical waiting time and provides laboratory evidence for making timely and correct clinical decisions.

Meanwhile, we plotted the correlation coefficient equation using the change in current and the number of GD2-positive cells, and the correlation coefficient was as high as 0.9532, which was helpful in clinically determining the presence or absence of GD2-positive cells in the bone marrow. In the clinic, because bone marrow aspiration is invasive and unrepeatable, obtaining trace amounts of bone marrow fluid and resuspending all the cells after centrifugation of each patient’s sample is subjected to a single flow-fluorescence assay for consistency; we measured the sample only once with the electrochemical biosensor. Similarly, in the clinic, physicians pay special attention to whether the cells are GD2-positive or -negative without knowing the exact amount because the mere presence of GD2-positive cells implies that the bone marrow has had metastases or relapses. The reason for the change in DPV in the bone marrow aspiration fluid from positive patients may be the production of antigen-antibody immune complexes leading to the functioning of the mass transfer and electron transfer blocking layer, which blocks the electron transfer channels and prevents the transfer of electrons to the metal surface [58,59].

Precisely detecting high-risk neuroblastoma bone marrow MRD is now a complex problem in clinical treatment [60]. Unfortunately, there are many limitations in obtaining bone marrow samples of high-risk NB, such as insufficient biopsy tumor cells, poor cooperation of the child in obtaining tumor tissue, or even inaccessibility [31,33,61]. In this study, we propose an innovative graphene/AuNP/GD2 Ab-functionalized electrode to obtain the electrochemical signal of individual cells for accurate counting of GD2-positive cells, which requires only 10 µL of sample volume and is equivalent to immunocytochemistry and flow cytometry, filling the gap for microscopic bone marrow specimens. This study demonstrates the promising application of the prepared electrochemical immunosensor in monitoring tumor metastasis.

## 5. Conclusions

Neuroblastoma, also known as the king of childhood tumors, is a rare solid tumor in children. High-risk neuroblastoma metastasizes easily and is difficult to treat. Due to its relatively low incidence, the development of clinical diagnostic technology has been slow and lagging behind, which is far from meeting clinical needs. In this study, we constructed an electrochemical detection platform for GD2-positive cell concentration, using GD2 antibodies to recognize GD2 molecules on the surface of individual cells, and then calculated the GD2-positive cell concentration to determine the presence of MRD in children with NB. The electrochemical signal was converted to cell concentration, significantly shortening the detection time. The traditional GD2 immunocytochemistry technique has the disadvantages of cumbersome operation, longer detection time, and more samples required, and the GD2 flow-fluorescence detection technique has the disadvantages of false-positive GD2 non-specific binding, false increase in the number of positive cells, and interference from GD2-positive cell debris. An graphene/AuNP/GD2 Ab-functionalized electrode effectively solves the drawbacks of traditional detection methods, is consistent with the efficacy of clinically recommended immunocytochemistry and flow cytometry results, and is expected to be widely used in clinical practice for the diagnosis of bone marrow MRD in high-risk NB. Meanwhile, a GD2 immunosensor provides a new idea for high-risk neuroblastoma POCT detection due to its easy operation and portability.

## Data Availability

The data presented in this study are available upon request from the corresponding author.
